# Oxytocin prolongs the gastric emptying time in patients with diabetes mellitus and gastroparesis, but does not affect satiety or volume intake in patients with functional dyspepsia

**DOI:** 10.1186/1756-0500-5-148

**Published:** 2012-03-16

**Authors:** Julia Borg, Bodil Ohlsson

**Affiliations:** 1 Department of Clinical Sciences, Division of Gastroenterology, Skåne University Hospital, Malmö, Lund University, Lund, Sweden

**Keywords:** Diabetes mellitus, Functional dyspepsia, Gastroparesis, Gastric emptying scintigraphy, Slow satiety drinking test

## Abstract

**Background:**

Oxytocin is released in response to a fatty meal. Blockage of the oxytocin receptor led to slower gastric emptying whereas stimulation resulted in less satiety in healthy volunteers. Patients with diabetes mellitus and gastroparesis lack oxytocin elevation, and dyspepsia is partly caused by reduced fundus accommodation causing early satiety and related symptoms. The aim of this study was thus to examine the effect of oxytocin on gastric emptying, satiety and volume intake in patients with gastrointestinal pathology.

**Results:**

Gastric emptying scintigraphy was performed twice in 12 patients with diabetic gastroparesis, once with oxytocin and once with saline as intravenous infusions. The patients scored their sensation of satiety using a visual analogue scale (VAS). The gastric emptying in patients with gastroparesis was prolonged during oxytocin infusion (*p *= 0.034) without affecting satiety. A slow satiety drinking test was performed in 14 patients with functional dyspepsia. The patients scored their satiety every five minutes until maximal satiety was reached, and the total volume was determined. The VAS was also completed 30 minutes afterwards. The test was performed twice, once with oxytocin and once with saline as intravenous infusions. There was no difference in satiety scores or volume of nutrient intake between saline and oxytocin infusions, either before, during or after the meal.

**Conclusions:**

Oxytocin prolongs gastric emptying in patients with diabetes mellitus and gastroparesis, but has no effect on volume of nutrient intake or satiety and other related symptoms in patients with functional dyspepsia.

## Background

Oxytocin and its receptor are expressed throughout the gastrointestinal (GI) tract [1,2]. Oxytocin is released in response to a fatty meal in healthy subjects [3], but patients with diabetes mellitus and gastroparesis have been found to lack this response [4]. Furthermore, administration of the oxytocin receptor antagonist atosiban delayed the gastric emptying in healthy subjects [5].

The effect of oxytocin on human gastric emptying has not been entirely established, with some studies showing an accelerated effect after a semisolid meal [6,7] and others showing no effect after a semisolid or solid meal [5,8]. In animal trials, studies have shown that oxytocin increases the gastric emptying time and induces satiety due to both central and peripheral effects [9-12]. On the other hand, one animal study has found that oxytocin increases gastric pressure and contracts gastric muscle fibers [13]. This dual effect may be explained by oxytocin exerting its effect both via cholecystokinin (CCK) release and CCK-receptors with subsequent inhibition of gastric emptying [11,12], and via oxytocin receptors stimulating gastric contraction [13].

The pathophysiology of dyspepsia is not yet completely known, but one theory is reduced ventricular accommodation in the fundus leading to various symptoms, e.g. early satiety. A slow drinking satiety test can be used to evaluate the accommodation and any possible pharmacological effects on accommodation and symptoms [14,15]. In a previous study in healthy subjects, we found that oxytocin reduced the sensation of satiety without affecting the volume of food intake or gastric emptying [8]. Seeing that functional dyspepsia and gastroparesis are the main clinical syndromes associated with gastric motor dysfunction [15], both of these disorders require further study in relation to oxytocin and its effects.

The aims of this study were to examine the effect of oxytocin 1) on the gastric emptying in patients with diabetes mellitus and gastroparesis, and 2) on satiety and volume intake in patients with functional dyspepsia.

## Results

### Gastric emptying test

**Figure 1:**
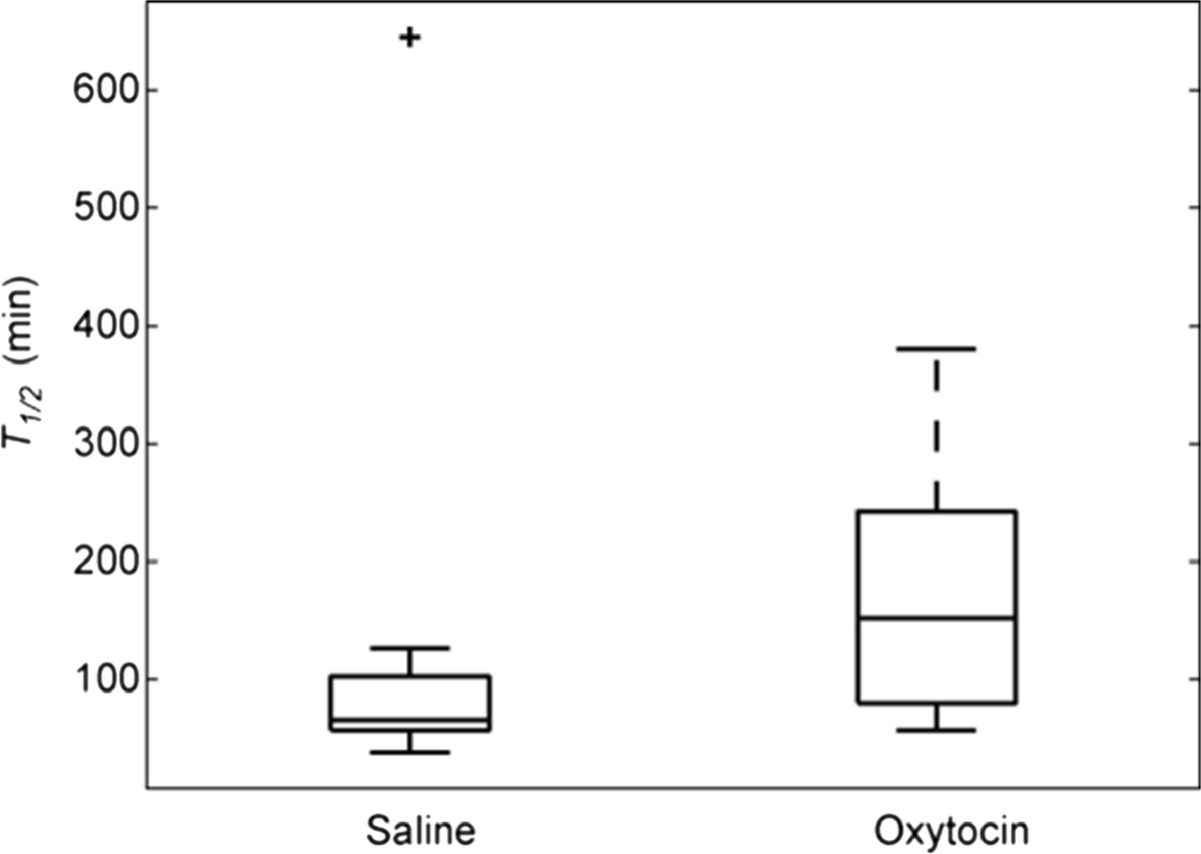
**Oxytocin infusions prolonged the gastric emptying time compared to saline in patients with diabetes mellitus and gastroparesis (*p *= 0.034)**. Values are given as box plots representing median (interquartile range). Wilcoxon's test.

The gastric emptying test showed that oxytocin prolonged the gastric emptying significantly compared to saline (Figure 1). Only one patient had longer gastric emptying time during saline than during oxytocin infusions. Although all patients included had verified gastroparesis during the year prior to inclusion, seven had a normal gastric emptying at the time of the saline trial, and three in the oxytocin trial. Three of the patients had normal gastric emptying in both trials.

**Figure 2:**
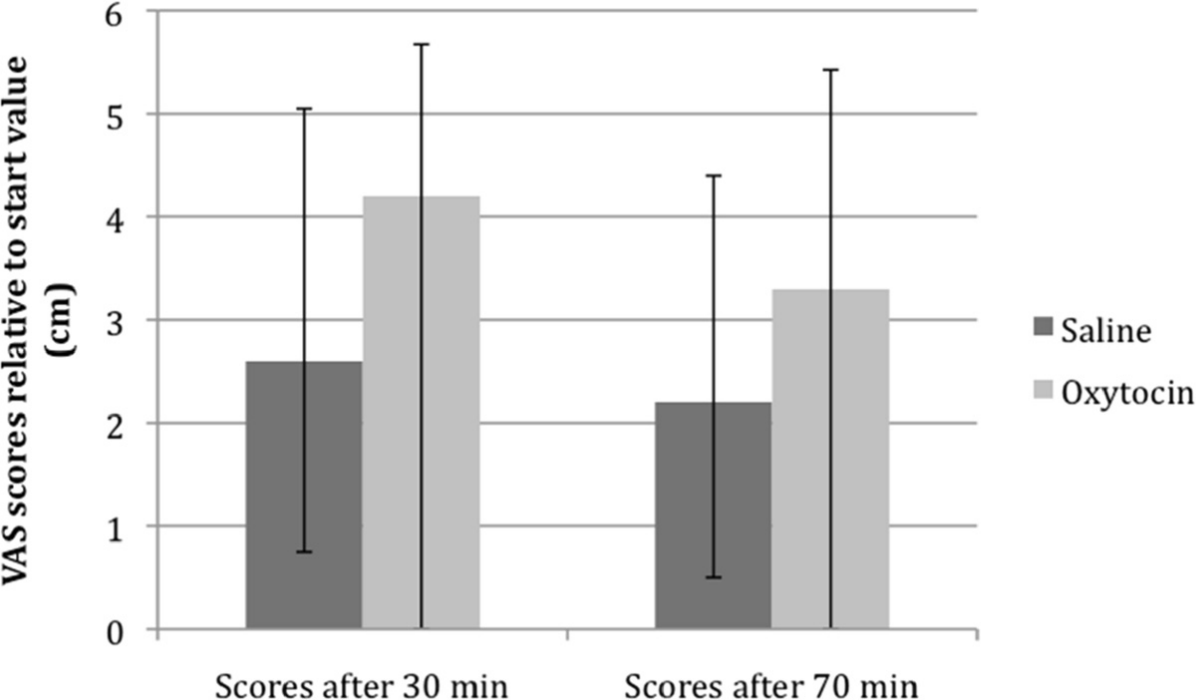
**There were no differences between saline and oxytocin in the VAS scores at 30 and 70 minutes after food intake during gastric emptying scintigraphy in patients with diabetes mellitus and gastroparesis (*p *= 0.594 respective *p *= 0.799)**. Values are given as boxplots representing median (interquartile range). Wilcoxon's test.

The fasting blood glucose values revealed no differences between the saline and oxytocin trials (Table 1). Also, the visual analogue scale (VAS) scores relative to start value, 30 and 70 minutes after food intake, did not differ between the two trials (Figure 2).

**Table 1:** Laboratory characteristics of patients with diabetes mellitus and gastroparesis

Parameter	Value
Diabetes duration (years)	32.0 (15.5-39.3)
BMI (kg/m^2^)	25.8 (22.4-30.0)
HbA1C (mmol/mol)	64.0 (59.0-68.8)
Fasting blood glucose, saline trial (mmol L^-1^)	9.3 (7.1-12.2)
Fasting blood glucose, oxytocin trial (mmol L^-1^)	9.6 (6.9-10.5)

BMI = body mass index, HbA1c = glycosylated hemoglobin. Values are given as median (interquartile range).

There was a statistically significant negative correlation between age and HbA1c (r = -0.68; *p *= 0.016), but no correlation between age and T1/2 for saline or oxytocin infusions (*p *= 0.639 and *p *= 0.687, respectively). Neither was there any tendency to correlation between HbA1c and T1/2 for saline or oxytocin infusions (*p *= 0.989 and *p *= 0.610, respectively).

### Satiety test

**Figure 3:**
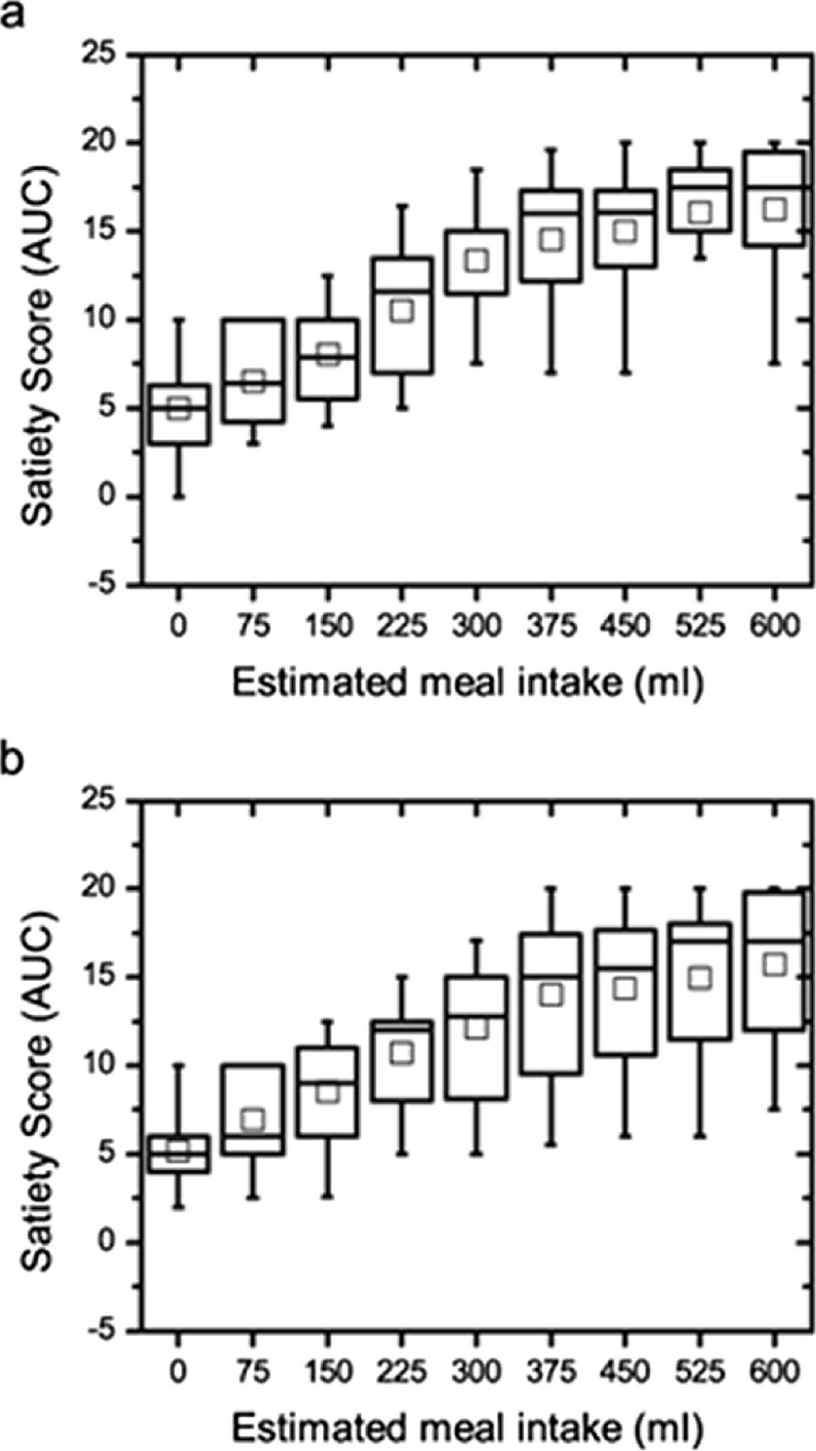
**The area under the curve (AUC) for the satiety scores between saline (a) and oxytocin (b) infusions in patients with functional dyspepsia did not differ (*p *= 0.953)**. Values are given as boxplots representing median (interquartile range). The square inside the boxplot represents mean value. Wilcoxon's test.

The satiety test showed no difference in the total volume of nutrient intake between the saline and the oxytocin infusions (*p *= 0.149). The satiety scores before, at maximal satiety and 30 minutes after finishing the meal did not differ between the saline and the oxytocin trials (*p *= 0.483, *p *= 0.449 and *p *= 0.450, respectively). Five patients stopped the test after 40 min. The area under curve (AUC) for the satiety scores up until this time was calculated, and no significant difference was seen (Figure 3; *p *= 0.953).

The degree of abdominal pain, nausea, abdominal fullness and bloating, registered in the VAS symptoms form 30 minutes after finishing the meal did not differ between the saline and oxytocin infusions (*p *= 0.735, *p *= 0.374, *p *= 0.674 and *p *= 0.735, respectively).

When dividing the group of patients with functional dyspepsia into those with and those without predominantly early satiety, no differences in volume of nutrient intake, satiety scores before, during and 30 minutes after, nor in the registered symptoms after the test were found (data not shown).

## Discussion

This study showed that oxytocin prolonged the gastric emptying time in patients with diabetes mellitus and gastroparesis, but did not affect the volume of nutrient intake in patients with functional dyspepsia. Oxytocin infusion did not result in lower satiety compared to saline infusion.

Gastroparesis is a disorder in which the gastric emptying is delayed without mechanical obstruction [16]. The pathophysiology is multifactorial, with the following pathologies recognized to date: vagal parasympathetic dysfunction, hyperglycemia, loss of enteric neurons, loss of neuronal nitric oxide expression, smooth muscle abnormalities and disruption of interstitial cells of Cajal networks [17-19].

Previous studies have shown that the oxytocin receptor antagonist atosiban prolonged gastric emptying in healthy subjects [5] and diabetic patients with gastroparesis lacked an elevation of the oxytocin plasma concentration postprandially compared to patients without gastroparesis [3,4]. These findings gave rise to the hypothesis that oxytocin may have an essential role in GI motility, especially gastric emptying. However, we could not see any effect of oxytocin on the gastric emptying [5,8]. This may be explained by the dual effect oxytocin evokes on normal GI physiology. First, a direct effect of oxytocin on its receptors leads to stimulation of gastric muscle contraction [13]. Secondly, as oxytocin stimulates to CCK release, an indirect action via CCK receptors are activated, leading to inhibition of gastric emptying [11,12]. As atosiban only blocks the oxytocin receptor, delayed gastric emptying was seen during the administration of this specific drug [5].

In the present study, oxytocin prolonged gastric emptying in diabetic gastroparesis. The previously observed lower oxytocin concentration in plasma in patients with gastroparesis must therefore be a secondary, and not a primary etiology of the delayed gastric emptying [4]. It is plausible that the oxytocin receptor pathway is destroyed before the CCK receptor pathway, leading to a loss of contractory stimulation by oxytocin but with a preserved inhibition by the CCK receptors [11-13]. Tension of the gastric wall gives rise to afferent signals to the brain [20]. As oxytocin further delays gastric emptying in a dysfunctional stomach, this may lead to a reduced oxytocin secretion from the hypophysis by a feedback mechanism involving the hypothalamus [21]. Oxytocin exerts both peripheral and central effects on the GI tract as it passes the blood brain barrier [22]. However, the latter pathway is not the primarily by which oxytocin affects gastric motility, which has been found when oxytocin and oxytocin antagonists have been given centrally [23]. It would be interesting to examine the effect of atosiban in gastroparesis, but this drug elevates blood glucose levels and could not be used on patients with diabetes mellitus for ethical reasons.

The effect of the CCK receptor antagonist loxiglumide, which blocks the inhibitory effects on gastric emptying [24], would also be an interesting drug to study in this context. In healthy subjects, an intravenous infusion of loxiglumide stimulated antral contraction and decreased the gastric emptying half time [25]. The R-isomer of loxiglumide, dexloxiglumide, has been tested on patients with functional dyspepsia [26] and functional constipation [27] but not on patients with gastroparesis. In functional dyspepsia, the CCK-receptor antagonist counteracted the increase in gastric volume and dyspeptic symptoms during duodenal lipid infusion, while also reducing gastric compliance [26]. However, the effect on functional constipation has been inconclusive [27].

Oxytocin has in a previous study been shown to be efficient in the treatment of functional abdominal pain and depressed mood [28]. This, together with the described analgesic effect by oxytocin in rats [29], make oytocin analogs to be promising drugs in the future treatment of irritable bowel syndrome (IBS) and depression. The present results of prolongation of gastric emptying by oxytocin underlines the importance of treating IBS patients with oxytocin or its newly developed analogs, first when GI dysmotility has been excluded.

Patients who have symptoms from the upper GI tract in the absence of any organic disease are diagnosed as suffering from functional dyspepsia [30]. Dyspepsia is one of the most common disorders in the GI tract. The findings of cellular pathology in dyspepsia are very sparse, among which a few studies have suggested an increased amount of gastric mast cells, eosinophil degranulation and afferent dysfunction [31,32]. Other studies have shown hypersensitivity in the GI tract to mechanical gastric distension, suggesting enhanced perception of physiological signals [33-35].

Reduced ventricular accommodation after meal intake has been described in 40% of patients with dyspepsia [36]. According to Tack et al. [37] reduced accommodation is associated with early satiety, making it an important symptom in this subgroup of patients. The slow drinking satiety test has been developed to measure ventricular accommodation to a meal and is therefore particularly suitable for studying this subgroup [14,15].

A previously performed study by our group showed that oxytocin reduces the sensation of satiety without affecting the volume of nutrient intake or gastric emptying in healthy subjects [8]. Thus, it could possibly affect early satiety and other symptoms in patients suffering from functional dyspepsia, why we performed the satiety test on these patients. Interestingly, we could not find a difference in satiety, volume intake, abdominal pain, nausea, abdominal fullness and bloating in these patients with oxytocin versus saline. Satiety was not either affected during the gastric emptying test. We have previously described that oxytocin decreases abdominal pain and depression in patients with functional disorders, but has no effect on constipation [28]. Thus, oxytocin seems to have some effects on sensory functions but is not efficient in the treatment of GI dysmotility and symptoms related to the dysmotility. The effect on abdominal pain in IBS may be explained by that the pain experience in IBS, and pain relief of oxytocin, are predominantly central effects [29,38], whereas effects rendering early satiety may predominantly be of peripheral characters [23,37].

One of the limitations of the present study is that although all patients were recently diagnosed as suffering from gastroparesis, some of them had normal gastric emptying in the present study. This may partly be explained by the variation in the GI motility from one day to another [39]. Further, hyperglycemia prolongs gastric emptying [40], and the patients may have been in better metabolic control at the time when they entered in the present study, than at the time of their last gastric emptying scintigraphy. Nevertheless, the patients cannot be considered to have normal gastric function as they have had clinical symptoms of dysfunction leading to admission for gastric scintigraphy, and have once been classified as gastroparetic by the same method. Another limitation is the small sample size, which depends on difficulties to recruite patients in a good metabolic control. The female predominance in the study depends on that these disorders are more common in women than in men [17,41].

## Conclusion

Oxytocin prolongs gastric emptying in patients with diabetes mellitus and gastroparesis, but has no effect on volume of nutrient intake or satiety and other related symptoms in patients with functional dyspepsia.

## Methods

The study has been performed according to the Declaration of Helsinki, and was approved by the Ethics Committee at Lund University and the Swedish Medical Agency (Dnr 2009/502). Written, informed consent was obtained from all participants. All of the subjects completed the tests and no adverse events were reported apart from a few patients suffering from headaches. Trial registration: NCT01152047, NCT00776360.

### Subjects

Patients with diabetes mellitus and gastroparesis, verified by an earlier gastric emptying scintigraphy during the previous year, were invited to undergo gastric emptying scintigraphy on two occasions, one with saline and one with oxytocin infusion, with at least 2 days in between. The patients were recruited from the Department of Endocrinology, Department of Clinical Sciences, Division of Gastroenterology and one primary health care centre in Malmö.

Exclusion criteria were age < 18 years or > 65 years and severe complications or cardiac symptoms, which may have entailed possible risks of side effects. Twelve patients (10 women), median age of 56.5 (52.0-64.8) years, in good metabolic control accepted to participate in the study. Glycosylated hemoglobin (HbA1c) was analysed at the Department of Chemistry, Skåne University Hospital, Malmö, according to clinical routines. HbA_1c _values were collected as Mono-S and subsequently converted to the National Glycohemoglobin Standardization Program (NGSP) standard by use of the following algorithm: 0.923 × HbA_1c _(Mono-S) × 1.345 = HbA_1c _(NGSP) [42]. Percentage HbA_1c _values were converted to the International Federation of Clinical Chemistry (IFCC) standard in mmol/mol according to the following equation: IFCC (mmol/mol) = ([NGSP (%)] - 2,152)/0.09148 [42].

Ten of 12 patients had other diabetic complications aside from gastroparesis, with retinopathy being the most common (Table 2). For further patient characteristics see Tables 1 and 2.

**Table 2:** Clinical characteristics of patients with diabetes mellitus and gastroparesis (n = 12)

	No (%)
Diabetes mellitus type 1	9 (75)
Diabetes mellitus type 2	3 (25)
Women	10 (83)
Men	2 (17)
Other complications of diabetes mellitus	10 (83)
- Retinopathy	8 (67)
- Peripheral neuropathy	7 (58)
- Nephropathy	2 (17)
- Other	7 (58)
Pharmacological treatment for gastrointestinal dysmotility	4 (33)
Insulin treatment	10 (83)
Oral hypoglycemic drugs	3 (25)
Hypothyroidism	3 (25)

Patients with functional dyspepsia were invited to perform a slow drinking satiety test on two occasions, one with saline and one with oxytocin infusion. All patients with dyspepsia fulfilled the Rome III criteria for functional dyspepsia [41]. They were recruited by advertisement or from a primary health care centre in Malmö. The most predominant symptoms were registered (Table 3). Exclusion criteria were age < 18 years or > 65 years, any other organic GI disorder or other severe diseases. Fourteen patients (12 women), median age of 37.5 (24.0-44.5) years, accepted to participate. None of the patients were using any drugs affecting GI motility.

**Table 3:** Clinical characteristics of patients with functional dyspepsia (n = 14)

	No (%)
Women	12 (86)
Men	2 (14)
Bloating as predominant symptom/symptoms	5 (36)
Early satiety as predominant symptom/symptoms	6 (43)
Epigastric pain as predominant symptom/symptoms	2 (14)
Epigastric burning as predominant symptom/symptoms	2 (14)

### Visual analogue scale (VAS)

The patients were asked to complete a questionnaire grading their hunger and/or feeling of satiety, using the VAS satiety scores graded from 0, for the most extreme hunger, to 20, for the most extreme satiety [5,8]. The VAS scale was labeled with different descriptions of hunger and satiety, from painful hunger to satiety combined with nausea, making it more illustrative. The patients' score at baseline was set to zero, and the values measured afterwards were set relative to this point. The scale was used in both the gastric emptying- and the slow drinking satiety test.

### Drugs

The subjects were examined on two different occasions and were given either an infusion of physiological saline or of oxytocin in random order during the experiments. Syntocinon^© ^(Novartis, Täby, Sweden), a synthetic analog of oxytocin, at a concentration of 8.3 μg mL^-1 ^was dissolved in 1000 ml saline and given as intravenous infusions for the duration of the experiment. The oxytocin infusion was given at concentrations of 80 and 40 mU min^-1^, respectively. These doses were chosen, as they were the most efficient in dose-response trials performed previously in healthy volunteers, and rendered pharmacological plasma concentrations of oxytocin [8]. Both the patients and the staff performing the gastric emptying and satiety tests were unaware of which infusion was being given.

### Gastric emptying test

The patients were studied in the morning after an overnight fast. Patients on regular medication influencing GI motility were asked to stop this treatment two days before the scintigraphy test. Blood glucose was measured before the start of the experiment. If b-glucose was > 18 mml L^-1 ^the experiment was stopped and the patient was sent home. When b-glucose was > 12 mmol L^-1^, the patients were given half of their ordinary insulin dosage at the laboratory before the start of the experiment.

A test meal was prepared by adding tin colloid labeled with 30-50 Mbq of ^99 m^Tc to an egg, which was whipped in a glass cup in a hot water bath until coagulated. The egg and a slice of toasted white bread were cut into pieces smaller than 1 × 1 cm and served with 100 ml 37°C water. The meal was eaten within five minutes. Immediately thereafter a large-field double-headed gamma camera (Philips Skylight, Philips Medical Systems, Best, The Netherlands) was placed anteriorly and posteriorly parallel to the upper abdominal wall. The radioactivity was measured continuously (1-min frames) for 70 minutes starting immediately after meal ingestion. A Region of Interest (ROI) representing the stomach was created and the activity of the first frame was taken as 100%. The gradual decreasing radioactivity, measured as the number of radioactivity decays per minute (counts/min), was plotted against time. The time elapsed to reach a 50% decrease of the activity in the ROI (T1/2) was identified as the point at which this plot crossed the 50% value. The values of the radioactivity measured were corrected for the half-life of ^99 m ^Tc, and for attenuation by using the geometrical mean values of the decay curves obtained from the two gamma camera heads used. T1/2 > 2 standard deviations (SD) for healthy control subjects (70 min) was considered abnormal [43].

An infusion of either saline or 80 mU oxytocin min^-1 ^was started at the same time as the meal intake began, and was given throughout the experiments. The subjects also scored their satiety using a VAS score, at time 0 when the meal intake began, and 30 and 70 minutes after the meal intake. The gastric emptying time and VAS scores were used for statistical calculations.

### Satiety test

The satiety test was performed according to a previously developed protocol by Tack et al. [14], using a slightly modified VAS scale which has been used in other studies performed by our group [5,8]. The patients were studied in the morning after an overnight fast. A peristaltic pump filled one of two beakers at a rate of 15 ml min^-1 ^with a liquid meal consisting of 13% protein, 48% carbohydrate and 39% lipids (Nutridrink^©^; Nutricia, Bornem, Belgium), giving 1.5 kcal ml^-1^. The patients were requested to maintain their intake at the filling rate, alternating the beakers as they were filled and emptied. The participants were instructed to terminate their meal when maximum satiety was reached and they could not continue to drink any more. At the start of the experiment and at five-minute intervals, they recorded their satiety using the VAS until they terminated their meal. A further VAS score for satiety and a separate VAS to score other GI symptoms, namely, abdominal pain, nausea, abdominal fullness and bloating, were completed 30 minutes after the end of the meal. An infusion of saline or 40 mU oxytocin min^-1 ^was started at the same time as the meal intake began and was terminated when the meal was ended. The volume of nutrient intake and registered VAS scores were used in the statistical calculations.

### Statistical analyses

Values are given as median (interquartile range, IQR). The VAS scores relative to baseline and AUC for VAS scores were calculated. Statistic differences in gastric emptying time and related VAS scores in the gastric emptying test and differences in satiety scores, AUC for VAS, volume of nutrient intake and VAS scores for GI symptoms in the satiety test were determined using Wilcoxon's test. Correlations were determined using Spearman's test. P < 0.05 was considered statistically significant.

## Abbreviations

AUC: Area under the curve; CCK: Cholecystokinin; GI: Gastrointestinal; HbA1c: Glycosylated hemoglobin; IBS: Irritable bowel syndrome; IFCC: International federation of clinical chemistry; NGSP: National glycohemoglobin standardization program; ROI: Region of Interest; SD: Standard deviation; T1/2: Time elapsed to reach a 50% decrease of the activity in the ROI; VAS: Visual analogue scale.

## Competing interests

The authors declare that they have no competing interests.

## Authors' contributions

Both authors designed the research study. BO contributed to essential reagents and tools. JB analyzed the data and wrote the paper. BO contributed to the manuscript with constructive criticism, and read and approved the final manuscript. All authors read and approved the final manuscript.
